# A closer look at the synthesis of 2-[^18^F]fluoroethyl tosylate to minimize the formation of volatile side-products

**DOI:** 10.1186/s41181-022-00179-8

**Published:** 2022-10-06

**Authors:** Martha Sahylí Ortega Pijeira, Sofia Nascimento dos Santos, Yasniel Babi Araujo, André Luis Lapolli, Marcio Nardelli Wandermuren, Zalua Rodríguez Riera, Ivone Carvalho, Philip H. Elsinga, Emerson Soares Bernardes

**Affiliations:** 1 Instituto de Pesquisas Energéticas e Nucleares (IPEN-CNEN/SP), São Paulo, SP CEP 05508-000 Brazil; 2 Radiotarget Biotecnologia Ltda, São Paulo, Brazil; 3 Chemistry Institute, University of São Paulo, São Paulo, SP CEP 05508-000 Brazil; 4 Departamento de Radioquímica, Instituto Superior de Tecnologías y Ciencias Aplicadas (InSTEC), Universidad de La Habana, 10400 Havana, Cuba; 5 School of Pharmaceutical Sciences of Ribeirão Preto, University of São Paulo (FCFRP–USP), Ribeirão Preto, CEP 14040-903 Brazil; 6 Department of Nuclear Medicine and Molecular Imaging, University Medical Center Groningen, University of Groningen, Groningen, Netherlands

**Keywords:** 2-[^18^F]Fluoroethyl tosylate, Radioactive gas, [^18^F]vinyl fluoride, 2-[^18^F]fluoroethanol, Radiation safety, PET tracers

## Abstract

**Background:**

2-[^18^F]Fluoroethyltosylate ([^18^F]FEtOTs) is a well-known ^18^F-fluoroalkylating agent widely used to synthesize radiotracers for positron emission tomography. The widespread use of [^18^F]FEtOTs is due in part to its low volatility when compared to other halide and sulfonate building blocks. In this work, the radioactive volatile side-products formed during the synthesis of [^18^F]FEtOTs were identified and characterized for the first time, and an optimization of the reaction conditions to minimize their formation was proposed.

**Results:**

In order to characterize the volatiles produced during [^18^F]FEtOTs synthesis, the reaction mixtures of both cold FEtOTs and [^18^F]FEtOTs were co-injected onto the HPLC system. The radioactive peaks corresponding to the volatile compounds were collected, analyzed through headspace gas chromatography mass spectrometry sampler (HS-GC–MS) and identified as vinyl fluoride ([^19^F]VF) and 2-fluoroethanol ([^19^F]FEOH). By using a rotatable central composite design with a two-level full factorial core of two factors (2^2^), it was determined that temperature and time are independent variables which affect the generation of [^18^F]VF and [^18^F]FEOH during the radiosynthesis of [^18^F]FEtOTs. In addition, in order to reduce the formation of the volatiles ([^18^F]VF and [^18^F]FEOH) and increase the yield of [^18^F]FEtOTs, it was demonstrated that the molar ratio of base to precursor must also be considered.

**Conclusion:**

[^18^F]VF and [^18^F]FEOH are volatile side-products formed during the radiosynthesis of [^18^F]FEtOTs, whose yields depend on the reaction time, temperature, and the molar ratio of base to precursor. Therefore, special care should be taken during the radiosynthesis and subsequent reactions using [^18^F]FEOTs in order to avoid environmental contamination and to improve the yield of the desired products.

**Supplementary Information:**

The online version contains supplementary material available at 10.1186/s41181-022-00179-8.

## Background

Positron emission tomography (PET) is a highly sensitive and non-invasive molecular imaging modality widely used in cardiology, neurology and oncology providing in vivo biochemical information Baâzaoui et al. (2016). Most of the PET imaging applications is performed with Fluorine-18 labeled radiopharmaceuticals due to the favorable nuclear and physical properties of this radionuclide. Fluorine-18 (^18^F) has a relatively short half-life (109.7 min), high positron branching ratio (97%), and low maximum energy of the emitted positron (0.635 MeV), which favors the resolution of PET images Jacobson et al. (2015).

**Fig. 1:**
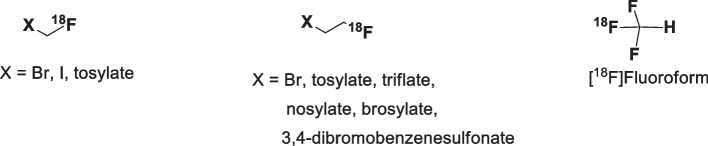
Short chain (n ≤ 2) ^18^Flabeled aliphatic building blocks (Born et al. 2017): [^18^F]Fluoromethyl bromide/ iodide/ tosylate, [^18^F]Fluoroethyl bromide/ tosylate/ triflate/ nosylate/ brosylate/ -3,4-dibromobenzenesulfonate, and [^18^F]Fluoroform

Several short chain (n ≤ 2) of ^18^F-labeled aliphatic building blocks have been used to prepare PET tracers (Fig. 1). Among them, 2-[^18^F]fluoroethyltosylate ([^18^F]FEtOTs) has been widely used because of its high reactivity towards nucleophilic substrates, good stability, low volatility, and easy purification in comparison to other volatile building blocks (Schoultz et al. 2013; Kniess et al. 2015; Born et al. 2017). In addition, [^18^F]FEtOTs is easily prepared by the nucleophilic ^18^F-substitution of the 1,2-ethylene ditosylate precursor. Table 1 indicates a selection of reports on the synthesis of [^18^F]FEtOTs showing a wide range of labeling temperatures (from 70 to 130 °C), reaction times (3 to 15 min) and base/precursor molar ratios (0.4 to 2.7).

**Table 1:** Selection of reported radiochemical yields of [^18^F]FEtOTs obtained by using a wide range of temperatures, reaction times, amounts of K_2_CO_3_ and OTs(CH_2_)_2_OTs, and base/precursor molar ratios

Entry	Temperature (°C)	Time (min)	K_2_CO_3_ (mg)	OTs(CH_2_)_2_OTs (mg)	Base/precursor (Molar ratio)	Yield (%)	References
1	70	15	1.7	8	0.6	62****	Erlandsson et al. 2009)
2	75	5	2	7.5	0.7	45***	Schoultz et al. 2013)
3	80	3	2	10	0.5	NR	Schirrmacher et al. 2002)
4	80	5	1.8	2	2.4	75*	Funke et al. 2012)
5	82	10	2.76	8.9	1	82***	Block et al. 1987)
6	85	3	2	4	1.3	70***	Bauman et al. 2011)
7	88	3	2	NR	-	60***	Riss et al. 2009)
8	90	3	2	4.5	1.2	84*	Tietze et al. 2006)
9	90	8	1	5	0.5	75–88**	Prenant et al. 2008)
10	90	10	0.7	5	0.4	65***	Wester et al. 1999)
11	95	5	2	8	0.7	35***	Sun et al. 2012)
12	95	10	2	5	1	75***	Majo et al. 2013)
13	100	10	5	5	2.7	90***	Li et al. 2012)
14	110	10	2	8	0.7	90**	Zheng et al. 2008)
15	125	5	0.7	5	0.4	NR	Elsinga et al. 2002)
16	130	4	2	5	1	NR	Shalgunov et al. 2015)

*NR* not reported

^*^ estimated by radio-HPLC analysis of the crude product

^**^ estimated by radio-TLC analysis of the crude product

^***^calculated at the end of the synthesis after product purification

^****^calculated at the end of the synthesis without product purification

Despite the wide application and advantages of [^18^F]FEtOTs, no report addressing the volatile side-products formed during [^18^F]FEtOTs synthesis has been published so far. Here we investigated how, and which volatile side-products are formed during the radiosynthesis of [^18^F]FEtOTs aiming a better understanding of the optimal conditions necessary to: (1) minimize the formation of volatile side-products from a radiation safety point of view; (2) improve the yield of the radiosynthesis of [^18^F]FEtOTs and; 3) improve the yield of the subsequent [^18^F]fluoroalkylation reactions.

## Methods

### General methods and instruments

All the chemicals and solvents were purchased with analytical grade from commercial sources and used without further purification. No-carrier-added [^18^F]F^−^ was produced by the ^18^O(p,n)^18^F reaction using enriched water (H_2_^18^O) as target material in an 18-MeV cyclotron (IBA, Belgium). Sep-Pak Light QMA cartridges were pre-conditioned with 10 mL of potassium carbonate 0.5 M (K_2_CO_3_), followed by 20 mL of Milli-Q water and purged with 20 mL air. µ-QMA cartridges were reused, and always flushed with 3 mL brine and 6 mL Milli-Q water before [^18^F]F^−^ trapping. Sep-Pak C18 Plus cartridges were preconditioned with 5 mL ethanol and 10 mL of Milli-Q water. Sep-Pak C18 Light cartridges were preconditioned with 3 mL ethanol and 3 mL of Milli-Q water. Radioactivity of [^18^F]F^−^ was measured using a Capintec radioisotope dose calibrator (CRC-15R, Ramsey, New Jersey, USA). High Performance Liquid-Chromatography (HPLC) system from Agilent Technologies (Santa Clara, CA, USA) was used for the LC analysis of the crude product. HPLC system are equipped with a model 1260 quaternary pump, a model 1260 UV absorbance detector, and a radioactivity detector from Raytest (Straubenhardt, Germany). The Agilent ChemStation software was used to operate the Agilent HPLC systems. The headspace sampler gas chromatography mass spectrometry (HS-GC–MS) equipment, model QP-2020, was manufactured by Shimadzu Corporation (Kyoto, Japan).

### Radiosynthesis of [^18^F]FEtOTs

**Scheme 1:**
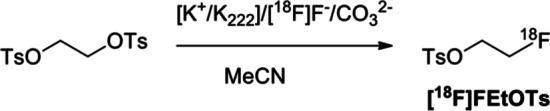
General scheme to prepare the [^18^F]FEtOTs following a bimolecular nucleophilic ^18^F-substitution-S_N_2-reaction

[^18^F]FEtOTs (Scheme 1) was manually prepared according to previous protocols (Schoultz et al. 2013; Schirrmacher et al. 2002). [^18^F]F^−^ was trapped onto a pre-conditioned QMA cartridge and was eluted with K_2_CO_3_ (2 mg, 14.5 µmol or 5 mg, 36.2 µmol) and Kryptofix 2.2.2 (K_222_, 11 mg, 29 µmol), dissolved in Milli-Q water (200 µL) containing MeCN (800 µL). [K^+^/K_222_]/[^18^F]F^−^/CO_3_^2−^ complex was dried by azeotropic distillation at 100 °C under nitrogen atmosphere with the sequential addition of MeCN (1 mL, 3 times). Then, the 1,2-ethylene ditosylate precursor (8 mg, 21.6 µmol) was dissolved in 1 mL of anhydrous MeCN and added to the vial containing the [K^+^/K_222_]/[^18^F]F^−^/CO_3_^2−^ dried complex (1.4 ± 0.3 GBq). Next, the vial was heated at different temperatures (from 70 to 130 °C) and time intervals (from 3 to 15 min) to optimize the reaction conditions. All radiochemical yields were determined by radio-HPLC analysis of the crude product, without further purification, unless stated otherwise.

### Synthesis of cold FEtOTs

The synthesis of non-radioactive FEtOTs was performed using sodium fluoride (NaF) as a source of fluoride anions and following the same protocol used for the radiosynthesis of [^18^F]FEtOTs. A solution of NaF (1 mg; 23.8 µmol) in Milli-Q water (1 mL) was loaded onto a QMA cartridge (carbonate form), and the trapped fluoride anions were eluted with K_2_CO_3_ (2 mg, 14.5 µmol) and Kryptofix 2.2.2 (11 mg, 29 µmol), dissoved in Milli-Q water (200 µL) and MeCN (800 µL) respectively. The azeotropic distillation was then performed at 100 °C under nitrogen atmosphere with a sequential addition of MeCN (1 mL, 3 times). Next, the 1,2-ethylene ditosylate precursor (8 mg, 21.6 µmol), dissolved in anhydrous MeCN (1 mL), was added to the vial containing the anhydrous fluoride anions. The vial was closed, and the reaction proceeded for 15 min at 130 °C. Finally, the reaction vial was cooled down at room temperature and stored at − 20 °C until further analysis.

### HPLC analysis

An aliquot of the crude mixtures was injected into the analytical HPLC using an Agilent Zorbax Eclipse Plus C18 5 µm 4.6 × 250 mm analytical column eluted with 55% of 0.1% trifluoroacetic acid (TFA) diluted in water (solvent A) and 45% of acetonitrile (MeCN) (solvent B) at 1 mL/min. The effluent was monitored with a UV absorbance detector set at 254 nm and a radioactivity detector.

### HS-GC–MS analysis

The samples were heated at 80 °C for 15 min for carrying out the headspace desorption. Then, 3-mL gas were aspirated from the vial with a syringe and directly injected onto the DB5-ms column (30 m × 0.25 mm i.d., 0.25-µm film thickness; Agilent Technologies) of the HS-GC–MS equipment. Helium 5.7 (99.97%) was used as carrier gas at a constant flow rate of 1.8 mL/min. Gradient analysis was run using the following temperature program: 40 °C (1 min); 40–150 °C (10 °C/min); 150–200 °C (50 °C/min); and 150 °C (3 min). The mass-to-charge ratio (m/z) ranged from 25 to 250 m/z.

### Purification and analysis of the non-radioactive volatile compounds by HPLC- and HS-GC–MS

To identify the radioactive signal(s) of the volatile side-product(s) in the radiochromatogram of [^18^F]FEtOTs, the radiolabeling of [^18^F]FEtOTs was performed as previously described with the following conditions: 15 min at 130 °C, using 2 mg of K_2_CO_3_ (14.5 µmol). In parallel, the synthesis of the non-radioactive FEtOTs was performed as described above. After cooling down, an aliquot of the non-radioactive FEtOTs reaction mixture was added to the reaction mixture of [^18^F]FEtOTs. The resulting mixture was injected onto the analytical HPLC using the same conditions as previously described. All peaks corresponding to the radioactive side-products were collected separately and stored at − 20 °C until the next day for radioactivity decay. Then, samples were analyzed by HS-GC–MS according to previously described procedures.

### Optimization of the radiosynthesis of [^18^F]FEtOTs

The influence of the labeling temperature [T(°C)] and the reaction time [t(min)] on the formation of radioactive volatile side-products was evaluated using a Rotatable Central Composite Design (RCCD) of response surface methodology (Ahirwar et al. 2016). A total of 12 set of experiments were conducted along with different combinations of the two independent variables T(°C) and t(min). The experimental RCCD had the four points of the two-level full factorial core 2^2^ (± 1), three center points (zero), four-star points located inside the studied range with an axial distance of 1.42 (± α), and one additional point (X). Table 2 summarizes the levels of the RCCD matrix. Unless otherwise specified, the ratio of base (K_2_CO_3_, 2 mg, 14.5 µmol) to precursor (1,2-ethylene ditosylate, 8 mg, 21.6 µmol) was chosen based on literature data (Sun et al. 2012; Zheng et al. 2008). The minimum (70 °C and 3 min) and maximum (130 °C and 15 min) parameters for T(°C) and t(min) were also chosen according to reported values for the radiosynthesis of [^18^F]FEtOTs found in the literature (Kniess et al. 2015). The experimental standard deviation was calculated with the set of experiments of the center points. The response variable was the yield of the characterized radioactive volatile side-product(s).

**Table 2:** Experimental rotatable central composite design (RCCD) matrix levels selected for temperature and time

Level	Temperature (°C)	Time (min)
− 1	70	3
+ 1	130	15
− α	79	5
+ α	121	13
0	100	9
X	100	10

± 1 minimum and maximum levels

± α star points in an axial distance of 1.42

0 center point

X additional point

### Effect of molar ratio of base to precursor during the radiosynthesis of [^18^F]FEtOTs

The molar ratio of base to precursor was evaluated at two different levels of labeling temperature (100 °C and 130 °C) and reaction time (1 min and 15 min), using two different molar masses of K_2_CO_3_ (2 mg, 14.5 µmol or 5 mg, 36.2 µmol) and keeping constant the amount of 1,2-ethylene ditosylate (8 mg, 21.6 µmol). The [^18^F]F^−^ was eluted from the QMA cartridge using K_2_CO_3_ (2 mg, 14.5 µmol or 5 mg, 36.2 µmol) and Kryptofix 2.2.2 (11 mg, 29 µmol), disoloed in Milli-Q water (200 µL) and MeCN (800 µL). All subsequent steps were performed as previously described.

### Statistical analysis

Data was expressed as the mean ± standard deviation. A p-value < 0.05 was considered statistically significant. The obtained data from the experimental RCCD plan was statistically processed by an analysis of variance (multifactor ANOVA) using the software STATGRAPHICS Plus *version 5.0*., to assess the effect of varying a single parameter (T (°C); t (min)), the simultaneous variation of both parameters (T (°C) x t (min)) and the presence of an optimal for temperature and/or time (T (°C) x T (°C); t (min) x t (min)) if p-value < 0.05.

## Results

### The radiosynthesis of [^18^F]FEtOTs leads to the formation of radioactive volatile side-products

**Fig. 2:**
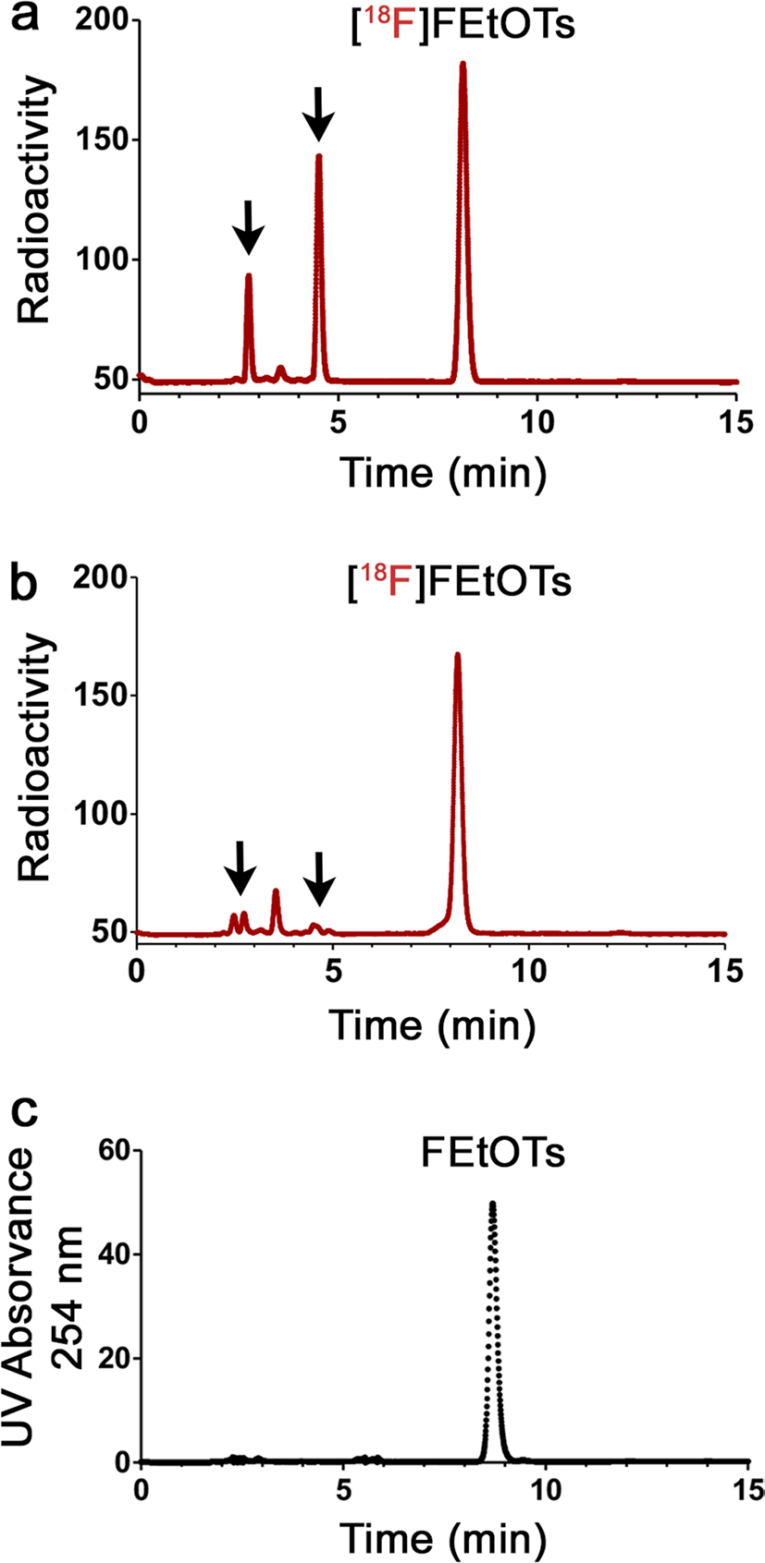
Radiochromatograms of the crude mixture of [^18^F]FEtOTs in **a** sealed (**b**) and unsealed reaction vials, and **c** chromatogram of the cold reference compound confirming the chemical identity of [^18^F]FEtOTs after analysis by analytical HPLC. The black arrows indicate the signals of the volatile side-products at 2.7 and 4.7 min on the radiochromatograms. The retention time of the cold reference FEtOTs was 8.7 min. HPLC chromatograms are representative of (n = 3) and were performed using an Agilent Zorbax Eclipse Plus C18 (5 µm, 4.6 × 250 mm) analytical column; elution with 0.1% TFA water / MeCN, 55:45 at 1 mL/min; monitored at 254 nm and with a radioactivity detector

During a common radiosynthesis of the ^18^F-fluoroalkylating agent [^18^F]FEtOTs using a sealed vial (130 °C, 15 min, and 2 mg of K_2_CO_3_), we observed the formation of two radioactive ^18^F-labeled side-products through HPLC analysis with retention times between 2.5 and 5 min (Fig. 2a). Corroborating this hypothesis, when the radiolabeling was performed in an unsealed vial (allowing the solvent to evaporate completely during the reaction), the radio-HPLC signals of 4.7 min and 2.7 min significantly decreased from 28% ± 2% to 2% ± 1% and from 11% ± 2% to 3% ± 1%, respectively (Fig. 2b). The formation of [^18^F]FEtOTs was always confirmed by comparing with the HPLC profile of the cold reference FEtOTs (Fig. 2c). These results suggest that radioactive volatile side-products are formed during the synthesis of [^18^F]FEtOTs.

### [^18^F]vinyl fluoride and 2-[^18^F]fluoroethanol are the radioactive volatile side-products formed during the radiosynthesis of [^18^F]FEtOTs

**Fig. 3:**
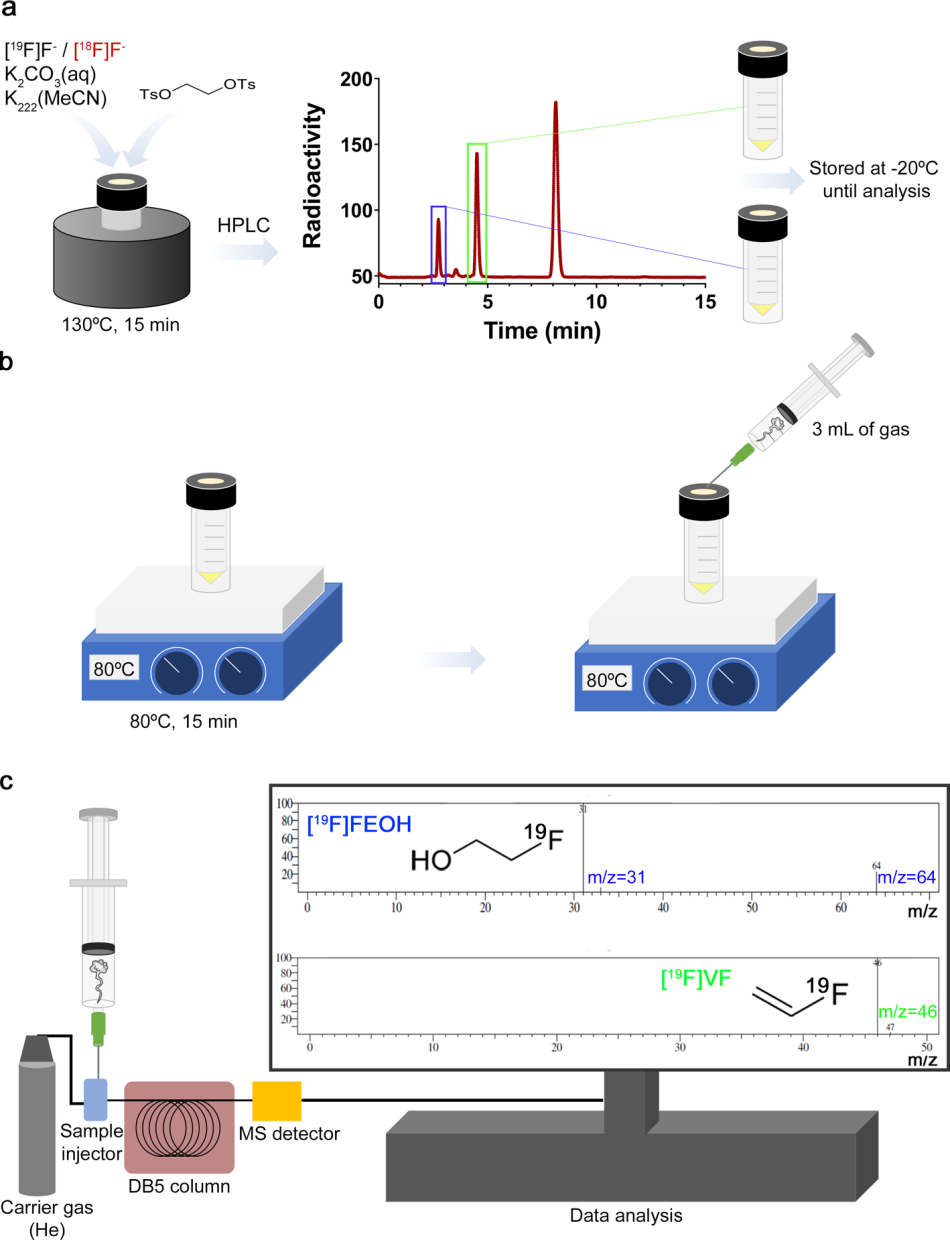
HS-GC–MS analysis of the cold FEtOTs crude reaction mixture. **a** [^18^F]FEtOTs and FEtOTs crude mixtures were co-injected and analyzed in an analytical HPLC using an Agilent Zorbax Eclipse Plus C18 (5 µm, 4.6 × 250 mm) analytical column; elution with 0.1% TFA water / MeCN, 55:45 at 1 mL/min; and monitored with a radioactivity detector. Fractions collected at 2.7 min and 4.7 min observed in the radio-HPLC chromatogram were frozen at − 20 °C until further analysis. **b** Samples were defrosted and heated at 80ºC, and 3 mL of gas was extracted from each collected sample. **c** Samples were analyzed by HS-GC–MS. Analysis showed the characteristic m/z ratios 31 and 64 corresponding to 2-fluoroethanol ([^19^F]FEOH), and m/z ratios 46 for vinyl fluoride ([^19^F]VF)

Since the quantity in moles of the side-products obtained during the synthesis of [^18^F]FEtOTs are minimal due to the very low amounts of ^18^F-activity used (1.4 GBq ~ 22 pmol), the synthesis of the cold compound FEtOTs was carried out using ^19^F (non-radioactive fluoride) and precursor in a 1:1 ratio (23.8 µmol: 21.6 µmol, respectively), which gives 10^6^ times more ^19^F in comparison to ^18^F (1.4 GBq). In order to better characterize the volatiles, both the reaction mixtures obtained after the radiosynthesis of [^18^F]FEtOTs and the non-radioactive reaction mixture of FEtOTs were combined and injected onto an analytical HPLC system. Next, the two radioactive compounds (at 2.7 and 4.7 min) were collected and stored at − 20 °C until further analysis (Fig. 3a). The collected samples were defrosted at room temperature, heated at 80 °C for 15 min (Fig. 3b) and then, a sample of the solvent vapors and gases were aspirated with a syringe-needle unit (3 mL) and injected directly into a HS-GC–MS system (Fig. 3c). A peak (m/z 31) corresponding to a fragment of CH_2_-OH was identified as 2-fluoroethanol in the fraction collected at 2.7 min ([^19^F]FEOH). In the same fraction, a second peak (m/z 64) was identified as the intact parental molecule for 2-fluoroethanol. In addition, commercially available 2-fluoroethanol was co-injected with the crude reaction mixture obtained during the synthesis of [^18^F]FEtOTs and further identified by HS-GC–MS in the radioactive fraction collected at 2.7 min (Additional file 1: Figure S1). Finally, a peak corresponding (m/z 46) to vinyl fluoride ([^19^F]VF) was identified in the fraction collected at 4.7 min. Altogether we identify [^18^F]VF and [^18^F]FEOH as volatile side-products formed during the synthesis of [^18^F]FEtOTs.

### The production of [^18^F]FEOH and [^18^F]VF are dependent on time and temperature

The influence of the radiolabeling temperature [T(°C)] and the reaction time [t(min)] in the formation of [^18^F]FEOH and [^18^F]VF were evaluated by using a rotatable central composite design (RCCD) with a two-level full factorial core for two factors (2^2^) (Ahirwar et al. 2016). The range of temperature (70 °C to 130 °C) and time (3 to 15 min), studied as independent variables, were selected based on reported data for the radiosynthesis of [^18^F]FEtOTs, and the molar ratio of base to precursor was fixed at 0.7. Ten different combinations of temperature and time were used for the radiosynthesis of [^18^F]FEtOTs (Table 3), with the combination 100 °C and 9 min (center points, 0) repeated three times to calculate the experimental standard deviation. The percentage of [^18^F]FEOH and [^18^F]VF were calculated from the radio-HPLC signals obtained from the crude mixture. The highest yield of [^18^F]FEOH (11%) and [^18^F]VF (28%) was observed when the radiolabeling method was conducted with the highest reaction temperature (130 °C) at the longest reaction time (15 min) (entry 4 of Table 3); whereas the lowest yield of [^18^F]FEOH (1%) and [^18^F]VF (2%) was obtained with the lowest reaction temperature (70 °C) at the shortest reaction time (3 min), (entry 1 of Table 3). On average, and considering all the parameters tested, the percentage of [^18^F]VF produced was about 3–4 times higher than the percentage of [^18^F]FEOH (Table 3).

**Table 3:** Yields of [^18^F]FEOH, [^18^F]VF and [^18^F]FEtOTs determined by radio-HPLC analysis of the crude product, obtained from the experimental plan of the rotatable central composite design

Entry	T (°C)	t (min)	[^18^F]FEOH (%)	[^18^F]VF (%)	[^18^F]FEtOTs (%)	[^18^F]VF/[^18^F]FEOH
1	70	3	1	2	61	2
2	70	15	5	16	74	3.2
3	130	3	7	26	60	3.7
4	130	15	11	28	60	2.6
5	100	9	7	23	59	3.3
6	100	9	5	20	61	4
7	100	9	5	18	62	3.6
8	100	10	5	23	59	4.6
9	79	9	2	13	62	6.5
10	121	9	5	20	52	4
11	100	5	3	11	67	3.7
12	100	13	8	25	58	3.1

Experimental standard deviation = 2. The base/precursor molar ratio was kept constant at 0.7

Accordingly, statistical analysis revealed that both temperature (p < 0.05) and time (p < 0.05) are positively correlated with the production of [^18^F]FEOH and [^18^F]VF (Table 4). Therefore, to minimize the production of [^18^F]FEOH and [^18^F]VF radioactive side-products during the radiosynthesis of [^18^F]FEtOTs, the balance between temperature and reaction time should be considered.

**Table 4:** P-values obtained from the variance analysis of [^18^F]FEOH and [^18^F]VF experimental yields at a 95% confidence

Effect	[^18^F]FEOH (p-value)	[^18^F]VF (p-value)
T (°C)	0.0020	0.0023
t (min)	0.0055	0.0177
T (°C) × t (min)	1.0000	0.1471
T (°C) × T (°C)	0.2430	0.5215
t (min) × t (min)	0.0963	0.8304

### Higher molar ratio of base to precursor improves the yield of [^18^F]FEtOTs

Although lower temperature and shorter reaction times lead to the lowest yields of the volatile side-products, these reaction conditions do not afford the best yield of [^18^F]FEtOTs. In order to improve the yield of radiosynthesis of [^18^F]FEtOTs and at the same time decrease the formation of the volatiles, the reaction time was shortened to one minute and the temperature set to 100 °C for further comparisons. In addition, we assessed the effect of increasing the molar ratio of base to precursor (from 0.7 to 1.7) on both the formation of volatiles and [^18^F]FEtOTs using two different reaction conditions: 130 °C for 15 min and 100 °C for 1 min.

**Fig. 4:**
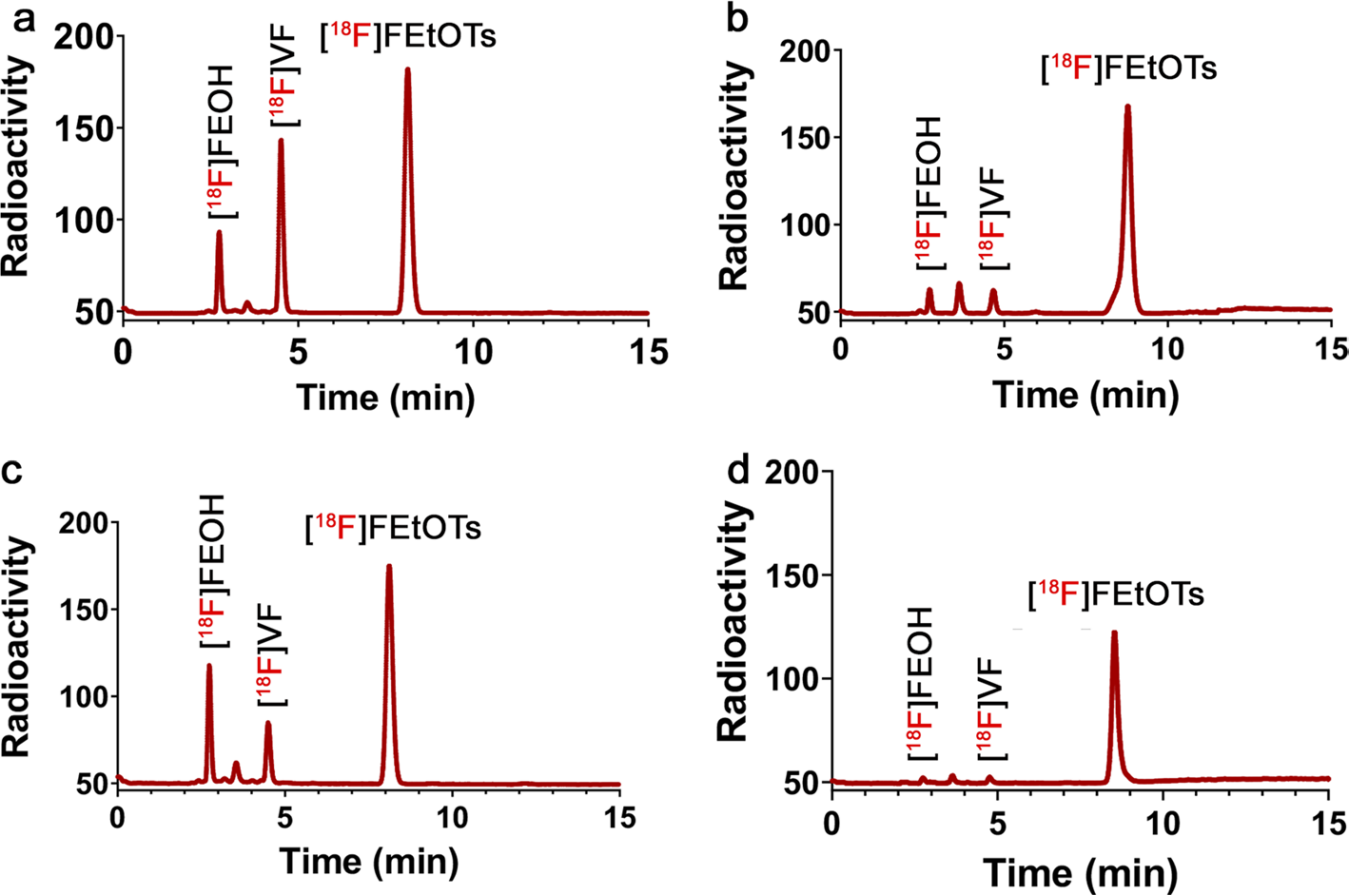
Radiochromatograms of crude mixtures of [^18^F]FEtOTs at two different conditions of time, temperature, and molar ratio of base to precursor. **a**: 130 °C—15 min—0.7. **b**: 100 °C—1 min—0.7. **c**: 130 °C—15 min—1.7. **d**: 100 °C—1 min—1.7. The retention times of [^18^F]FEOH and [^18^F]VF were 2.7 and 4.7 min, respectively. The retention time of the cold reference FEtOTs was 8.7 min. HPLC chromatograms are representative of (n = 3) and were performed using an Agilent Zorbax Eclipse Plus C18 (5 µm, 4.6 × 250 mm) analytical column; elution with 0.1% TFA water / MeCN, 55:45 at 1 mL/min; monitored with a radioactivity detector

When the radiosynthesis was performed at 130 °C for 15 min and the molar ratio of 1.7 (base to precursor), there was a decrease of 50% in the yield of [^18^F]VF (12% ± 4%), whereas the yield of [^18^F]FEOH almost doubled (19 ± 4%) in comparison with the radiosynthesis performed at the same condition but using the molar ratio of 0.7 (base to precursor) (Fig. 4a and 4c and entry 1 and 2 from Table 5). Interestingly, both the highest yield of [^18^F]FEtOTs and the lowest yield of [^18^F]VF were obtained when using the molar ratio of 1.7 (base to precursor) at 100 °C for 1 min (Fig. 4d compared to 4b using the molar ratio of 0.7 and entries 4 and 3 from Table 5, respectively). Surprisingly, the yield of [^18^F]FEOH increased when the radiosynthesis was performed at 130 °C for 15 min using the molar ratio of 1.7 (Fig. 4a and 4c and entry 1 and 2 from Table 5). Overall, these results suggest that besides controlling temperature and reaction time, the molar ratio of base to precursor plays an important role in order to control the formation of volatiles side-products formed during the radiosynthesis of [^18^F]FEtOTs.

**Table 5:** Yields of [^18^F]FEOH, [^18^F]VF and [^18^F]FEtOTs determined by radio-HPLC analysis of the crude product, obtained at different temperature, time and the base/precursor molar ratio

Entry	T (°C)	t (min)	K_2_CO_3_ (mg)	Base/precursor (Molar ratio)	[^18^F]FEOH (%)	[^18^F]VF (%)	[^18^F]FEtOTs (%)	[^18^F]VF/[^18^F]FEOH
1	130	15	2	0.7	10 ± 2	29 ± 2	59 ± 2	2.9
2	130	15	5	1.7	19 ± 4	12 ± 4	65 ± 4	0.6
3	100	1	2	0.7	3 ± 1	6 ± 2	80 ± 4	2
4	100	1	5	1.7	3 ± 1	2 ± 1	91 ± 1	0.7

Experiments were performed in triplicates

## Discussion

Many PET tracers have been synthesized using [^18^F]FEtOTs since its first radiosynthesis reported in 1987 (Block et al. 1987). Because of its favorable properties, [^18^F]FEtOTs has been widely used in ^18^F-fluoroalkylation reactions with nucleophilic substrates, displaying high reactivity as phenolic, thiophenolic, carboxylic and amine functionalities (Kniess et al. 2015). Moreover, [^18^F]FEtOTs has a good balance between the reactivity of the tosylate leaving group, hydrolytic stability, and a relatively low volatility (Schoultz et al. 2013). The low volatility of [^18^F]FEtOTs makes it more applicable to automation than its halide analogue and, in comparison to the [^18^F]fluoromethylated building block, [^18^F]fluoroethylated tracers have shown higher in vivo stability (Born et al. 2017). Despite its wide application and advantages of [^18^F]FEtOTs, no report addressing the side products formed during [^18^F]FEtOTs synthesis has been published so far. In the present manuscript, we identified two volatile molecules ([^18^F]FEOH and [^18^F]VF) formed during [^18^F]FEtOTs synthesis. In addition, in order to improve the yield and reduce the formation of these volatiles, we performed a series of experiments to determine the effect of temperature, time and the molar ratio of base to precursor on the yield of both product ([^18^F]FEtOTs) and volatile side-products ([^18^F]FEOH and [^18^F]VF).

[^18^F]FEtOTs has been prepared by others through nucleophilic ^18^F-substitution on the 1,2-ethylene ditosylate precursor using different reaction temperature and time, solvent volume, and base/precursor molar ratios, as some examples were summarized in Table 1. In general, reaction times varied between 3 and 15 min, and base/precursor molar ratios ≥ 1 exhibit better yields compared to the reactions carried out in less basic conditions, which is an important aspect to take into account. However, the authors have used different procedures to determine the reaction yield (such as radio-HPLC or radio-TLC analysis) calculated at the end of the synthesis or in the crude product, which could account for the differences in [^18^F]FEtOTs calculated yields. According to our results (Table 3), the formation of both side-products ([^18^F]VF and [^18^F]FEOH) is favored by higher temperatures (≥ 100 °C) and the reaction times higher than 3 min, keeping constant the base/precursor ratio at 0.7. In addition, an increase in the reaction time (from 3 to 15 min) also favored the formation of [^18^F]VF and [^18^F]FEOH even at the lower temperature (70 °C).

It is broadly discussed in the literature how harsh basic conditions might lead to degradation of precursors and reaction products leading to formation of side-products (Jacobson et al. 2015; Kniess et al. 2015; Carberry et al. 2011; Krasikova and Orlovskaya 2022). In this regard, Fedorynski and coworkers (Fedorynski et al. 1978) used a combination of sodium and/or potassium carbonates that alongside with crown ethers formed strong bases in organic solvents for generation and reactions of a variety of carbanions. This led us to believe that the combination of both K_222_/K_2_CO_3_ with a high base/precursor ratio (> 1.5) could create a strong basic environment in the reaction mixture. In fact, when the base/precursor ratio increased from 0.7 to 1.7 at the same reaction temperature and time (Table 5), we could observe a reduction in [^18^F]VF yield, and increased [^18^F]FEOH yield, suggesting that the latter is favored in a more basic medium, increased temperature (> 100 °C) and reaction times (> 3 min). In a less basic medium, on the other hand, the thermal decomposition of both 1,2-ethylene ditosylate and the product [^18^F]FEtOTs is favored, leading to the formation of [^18^F]VF and potassium *p*-toluenesulfonate. Corroborating our data, it has been demonstrated that sulfonate ester linkage containing polymers may be decomposed by thermal treatment at 90-180 °C under acidic conditions, releasing sulfonic acid and vinyl compounds (Ma and Webster 2018; Ito and Ueda 1988).

There are no reports in the literature, so far, addressing the mechanism by which [^18^F]FEOH is formed during [^18^F]FEtOTs “conventional” radiosynthesis using the K_2_CO_3_/K_222_ system in dry acetonitrile. Still, Shinde et al., (Shinde et al. 2021) showed that the synthesis of [^18^F]fluoropropyl tosylate using 1,3-ditosylpropane as precursor and a base/precursor molar ratio of 1 in dry conditions, generated a side-product (29% yield) that was identified as [^18^F]fluoropropanol. Furthermore, the same synthesis was performed using tetraethyl ammonium bicarbonate (TEAB) and tri-(tert-butanol)-methylammonium iodide (TBMA-I) as phase transfer catalysts, with 60% RCY of [^18^F]fluoropropyl tosylate and 7% of [^18^F]-fluoropropanol for TEAB; and 21% RCY of [^18^F]fluoropropyl tosylate with no formation of [^18^F]fluoropropanol when using TBMA-I. The authors concluded that TBMA-I is less basic compared to the K_2_CO_3_/K_222_ system and that the coordination of [^18^F]fluoride with TBMA-I prevented the formation of [^18^F]fluoropropanol. In our study, using the K_2_CO_3_/K_222_ system we observed that an increase in base/precursor molar ratio (from 0.7 to 1.7) resulted in a twofold increase in the amount of [^18^F]FEOH, when the reaction was performed at 130 °C for 15 min (Table 5).

Despite the widespread use, to the best of our knowledge, this is the first report to identify and characterize the radioactive volatile side-products formed during the radiosynthesis of [^18^F]FEtOTs.

Neal and coworkers (Neal et al. 2005) suggested that [^18^F]tosyl fluoride (a moderately volatile compound) was formed as side-product during the synthesis of [^18^F]fluoromethyl tosylate. These authors assessed the influence of different solvents (acetonitrile, dimethylformamide and acetone) using ^18^F- solubilized with different mixtures (tetrabutylammonium bicarbonate, K_222_/K_2_CO_3_ and K_222_/KHCO_3_), and different amounts of complexing agent Kryptofix and bis(tosyloxy) methane precursor; still, none of the tested modifications improved the yield of [^18^F]fluoromethyl tosylate (Neal et al. 2005). Nonetheless, they discovered that the addition of a small amount of water to the reaction significantly increased the yield of [^18^F]fluoromethyl tosylate and proposed that the effect of water on the reaction was due to (1) the fluoride liberated from the selective hydrolysis of [^18^F]tosyl fluoride, which would be then available to react with bis(tosyloxy) methane precursor driving the reaction toward the formation of [^18^F]fluoromethyltosylate: and (2) the increased solubility of the hydrated fluoride ion complex (Neal et al. 2005). Although we have not assessed the effect of water in our reaction settings, we cannot discard that [^18^F]tosyl fluoride can also be formed during the synthesis of [^18^F]FEtOTs through the nucleophilic attack of [^18^F]fluoride on the sulfur atom of [^18^F]FEtOTs, yielding both [^18^F]tosyl fluoride and [^18^F]FEOH. Indeed, we may have overlooked the formation of [^18^F]tosyl fluoride because we did not measure the amount of radioactivity that remained stuck in the reaction vessel nor the recovery of radioactivity from the HPLC analytical column, both of which represent the main limitations of our study.

Interestingly, two previous reports had suggested the formation of 2-[^18^F]fluoroethanol and vinyl fluoride during the synthesis of [^18^F]FEtOTs and [^18^F]fluoroalkylation reaction using [^18^F]FEtOTs, respectively (Tietze et al. 2006; Vries et al. 2003). Although Tietze et al. 2006 (Tietze et al. 2006) reported a decreased production of what they supposed to be 2-[^18^F]fluoroethanol when the reaction time was shortened to 3 min at 90 °C and using a base/precursor ratio of 1.25, the chemical structure of this compound was not characterized. In addition, de Vries et al. 2003 (Vries et al. 2003) suggested that [^18^F]VF is formed during the [^18^F]fluoroethylation of target molecules using [^18^F]FEtOTs after heating to 90 °C for 15 min. Hence, the formation of radioactive gas is expected both during the radiosynthesis of [^18^F]FEtOTs and the subsequent [^18^F]fluoroalkylation reaction. Although in the present manuscript we have addressed only the reaction conditions that affect the formation of [^18^F]FEtOTs and its volatiles side-products, we demonstrated here that higher temperatures (Table 3) and longer reaction time (> 3 min) have the potential to also affect the subsequent alkylation reactions using [^18^F]FEtOTs. In addition, special care should be taken to avoid the potential risk of inhalation of radioactive gases during the radiosynthesis of [^18^F]FEtOTs and the subsequent [^18^F]fluoroalkylation reaction.

## Conclusions

We characterized for the first time two volatiles side-products formed during [^18^F]FEtOTs synthesis, which were identified as 2-[^18^F]fluoroethanol and [^18^F]vinyl fluoride. The increase of labeling temperature and reaction time significantly enhanced the formation of these radioactive gases. On the other hand, we can increase the yield of [^18^F]FEtOTs and reduce the formation of 2-[^18^F]fluoroethanol and [^18^F]vinyl fluoride by controlling time, temperature, and the molar ratio of base to precursor.

## Supplementary Information

**Additional file 1: Figure S1**. Results of the HPLC and HS-GC-MS analyses of the [^18^F]FEtOTs crude mixture co-injected with commercially available 2-fluoroethanol (at m/z 64 according to our HS-GC-MS results) after heating at 130oC for 15 min and using 2 mg of K_2_CO_3_.

## Data Availability

Contact the corresponding author.

## Abbreviations

[^18^F]F^−^: ^18^F-fluoride
[^18^F]FEOH: 2-[^18^F]fluoroethanol
[^18^F]FEtOTs: 2-[^18^F]fluoroethyl tosylate
^18^F: Fluorine-18
[^18^F]FV: [^18^F]vinyl fluoride
HS-GC–MS: Headspace sampler gas chromatography mass spectrometry
HPLC: High pressure liquid chromatography
K_222_: Kryptofix 2.2.2
K_2_CO_3_: Potassium carbonate
KHCO_3_: Potassium bicarbonate
m/z: Mass-to-charge ratio
MeCN: Acetonitrile
NaF: Sodium fluoride
PET: Positron emission tomography
QMA: Quaternary methyl ammonium
RCCD: Rotatable central composite design
TFA: Trifluoroacetic acid
TLC: Thin layer chromatography
