# Supplementary material for: A closer look at the synthesis of 2-[18F]fluoroethyl tosylate to minimize the formation of volatile side-products
Source: EJNMMI Radiopharm Chem. 2022 Oct 6;7:26. doi: 10.1186/s41181-022-00179-8 (PMC9537402; doi:10.1186/s41181-022-00179-8)
Supplement: Supplementary file 1 — Additional file 1: Figure S1. Results of the HPLC and HS-GC-MS analyses of the [18F]FEtOTs crude mixture co-injected with commercially available 2-fluoroethanol (at m/z 64 according to our HS-GC-MS results) after heating at 130oC for 15 min and using 2 mg of K2CO3. [file 41181_2022_179_MOESM1_ESM.docx]

**A closer look at the synthesis of 2-[^18^F]fluoroethyl tosylate to minimize the formation of volatile side-products**

Martha Sahylí Ortega Pijeira^1^, Sofia Nascimento dos Santos^1,2^, Yasniel Babi Araujo^1^, André Luis Lapolli^1^, Marcio Nardelli Wandermuren^3^, Zalua Rodríguez Riera^4^, Ivone Carvalho^5^, Philip H. Elsinga^6^, and Emerson Soares Bernardes^1,^*

^1^Instituto de Pesquisas Energéticas e Nucleares (IPEN-CNEN/SP), CEP 05508-000 São Paulo, SP, Brazil; [msopijeira@gmail.com](mailto:msopijeira@gmail.com%20), [snsantos@alumni.usp.br](mailto:snsantos@alumni.usp.br), [yasniel@usp.br](mailto:yasniel@usp.br), [alapolli@ipen.br](mailto:alapolli@ipen.br), [ebernardes@ipen.br](mailto:ebernardes@ipen.br)

^2^ Radiotarget Biotecnologia Ltda, São Paulo, Brazil

^3^ Chemistry Institute, University of São Paulo, CEP 05508-000 São Paulo, SP, Brazil; [nardelli@iq.usp.br](mailto:nardelli@iq.usp.br)

^4^ Departamento de Radioquímica, Instituto Superior de Tecnologías y Ciencias Aplicadas (InSTEC), Universidad de La Habana, CP 10400, La Habana, Cuba; [zalua@instec.cu](mailto:zalua@instec.cu)

^5^ School of Pharmaceutical Sciences of Ribeirão Preto, University of São Paulo (FCFRP–USP), CEP 14040-903 Ribeirão Preto, Brazil; [carronal@usp.br](mailto:carronal@usp.br)

^6^ Department of Nuclear Medicine and Molecular Imaging, University Medical Center Groningen, University of Groningen, Groningen, Netherlands; [p.h.elsinga@umcg.nl](mailto:p.h.elsinga@umcg.nl)

***** Corresponding author: ebernardes@ipen.br; Tel.: +55-11-31339546

**Supplementary information**

**Figure S1**

**
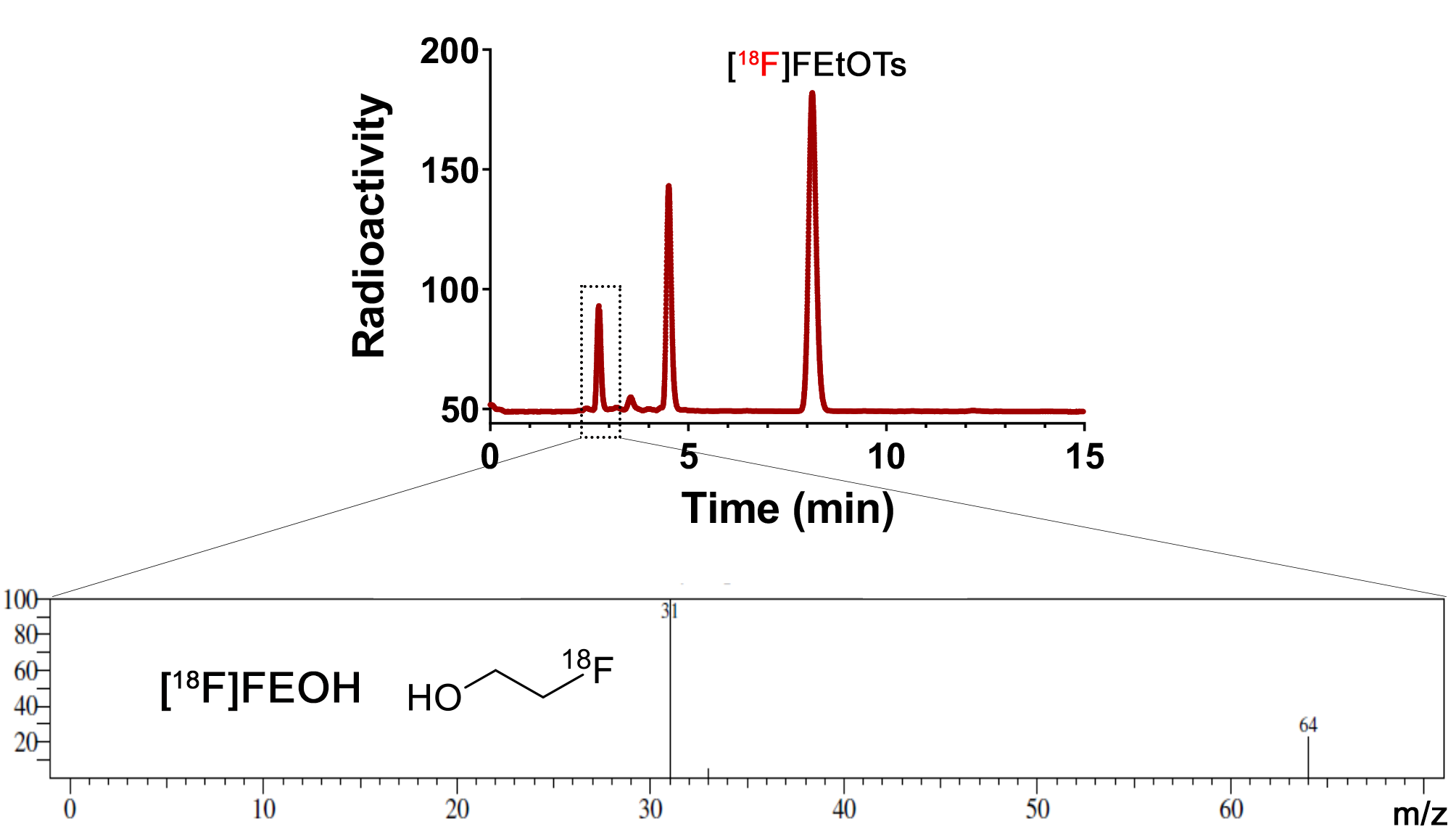
**

**Figure S1.** Results of the HPLC and HS-GC-MS analyses of the [^18^F]FEtOTs crude mixture co-injected with commercially available 2-fluoroethanol (at m/z 64 according to our HS-GC-MS results) after heating at 130^o^C for 15 min and using 2 mg of K_2_CO_3_.

**Supplementary Material and methods**: Co-analysis of [^18^F]FEtOTs reaction mixture co-injected with the standard compound 2-fluoroethanol by HPLC and HS-GC-MS
